# Advances in metal graphitic nanocapsules for biomedicine

**DOI:** 10.1002/EXP.20210223

**Published:** 2022-03-15

**Authors:** Shengkai Li, Yanxia Yang, Shen Wang, Yang Gao, Zhiling Song, Long Chen, Zhuo Chen

**Affiliations:** ^1^ Molecular Science and Biomedicine Laboratory (MBL) State Key Laboratory of Chemo/Bio‐Sensing and Chemometrics College of Chemistry and Chemical Engineering Aptamer Engineering Center of Hunan Province Hunan Provincial Key Laboratory of Biomacromolecular Chemical Biology Hunan University Changsha China; ^2^ College of Materials Science and Engineering Hunan Province Key Laboratory for Advanced Carbon Materials and Applied Technology Hunan University Changsha China; ^3^ Key Laboratory of Optic‐Electric Sensing and Analytical Chemistry for Life Science MOE Shandong Key Laboratory of Biochemical Analysis College of Chemistry and Molecular Engineering Qingdao University of Science and Technology Qingdao China; ^4^ Faculty of Science and Technology University of Macau Macau SAR China

**Keywords:** biodetection, bioimaging, chemical vapor deposition, metal graphitic nanocapsules, therapy

## Abstract

Metal graphitic nanocapsules have the advantages of both graphitic and metal nanomaterials, showing great promise in biomedicine. On one hand, the chemically inert graphitic shells are able to protect the metal core from external environments, quench the fluorescence signal from the biological system, offer robust platform for targeted molecules or drugs loading, and act as stable Raman labels or internal standard molecule. On the other hand, the metal cores with different compositions, sizes, and morphologies show unique physicochemical properties, and further broaden their biomedical functions. In this review, we firstly introduce the preparation, classification, and properties of metal graphitic nanocapsules, then summarize the recent progress of their applications in biodetection, bioimaging, and therapy. Challenges and their development prospects in biomedicine are eventually discussed in detail. We expect the versatile metal graphitic nanocapsules will advance the development of future clinical biomedicine.

## Graphical abstract

In this review, we summary the preparation, classification, and properties of versatile metal graphitic nanocapsules, and then introduce the recent progress of their applications in biodetection, bioimaging, and therapy in detail. In addition, challenges and their development prospects in biomedicine are also discussed. We expect the metal graphitic nanocapsules will advance the development of future clinical biomedicine.

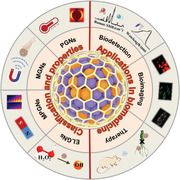

## 1 INTRODUCTION

Graphitic nanomaterials have many outstanding advantages including large specific surface area, easy functionalization, excellent fluorescence quenching performance, good biocompatibility, and large Raman scattering cross section.^[^ ^1^ ^]^ To broaden their applications in biomedicine, great attention has been focused on integrating graphitic materials with other inorganic nanomaterials, resulting in the development of novel metal–graphitic hybrid nanomaterials.^[^ ^2^ ^]^ Metal–graphitic hybrid nanomaterials have the merits of both graphitic and metal nanomaterials, thus showing broader application prospects.^[^ ^3^ ^]^ Nevertheless, metal–graphitic hybrid nanomaterials show poor stability in harsh environments such as long‐term laser irradiation, high‐concentration salt solutions, or strong acidic environment, which probably lead to bioanalytical inaccuracy, reduced therapeutic effect, and even unexpected biotoxicity.^[^ ^4^ ^]^

Metal graphitic nanocapsules, a new type of graphitic nanomaterials with the metal cores confined in the nanospace of few‐layer graphitic shell, show superior stability in harsh conditions The intact function of the metal cores is maintained without the interference of the external environment due to the isolation of chemically inert graphitic shell, thus laying a solid foundation for their biomedical applications.^4^, ^5^ ^]^ Currently, metal graphitic nanocapsules with different compositions, sizes, and morphologies have been prepared by simple chemical vapor deposition (CVD) method.^4^, ^5^ ^]^ According to the composition and properties of the metal core, metal graphitic nanocapsules are divided into plasmonic graphitic nanocapsules (PGNs), magnetic graphitic nanocapsules (MGNs), magnetic‐plasmonic graphitic nanocapsules (MPGNs), and enzyme‐like graphitic nanocapsules (ELGNs), and their extensive biomedical applications involve in biodetection, bioimaging and therapy are systematically introduced.

First, metal graphitic nanocapsules have been used for surface enhance Raman scattering (SERS), fluorescence, and colorimetric bioanalysis. Benefiting from the superior SERS activity of the plasmonic core, PGNs and MPGNs have been used for SERS analysis.^[^ ^6^ ^]^ Significantly, their graphitic shell with unique Raman scattering characteristic bands acts as the internal standard (IS) molecule, avoids the direct contact between the inner metal core and analytes or external environment, and minimizes photocarbonization of analytes and unnecessary reactions, which further improves the accuracy of SERS bioanalysis.^6^, ^7^ ^]^ Benefiting from the separation, enrichment ability of the magnetic core, and the fluorescence quenching performance of the graphitic shell, MGNs have been used for fluorescence bioanalysis.^[^ ^8^ ^]^ Besides, ELGNs with superior enzyme‐like activity has been used for colorimetric bioanalysis.^[^ ^9^ ^]^

Second, metal graphitic nanocapsules have been used for Raman imaging, two‐photon luminescence (TPL) imaging, and magnetic resonance imaging (MRI). The unique Raman scattering bands of the graphitic shell act as the stable Raman tags, especially the 2D band located in the cellular silent region (1800–2800 cm^−1^) avoids the interference from environment, in vivo/in vitro Raman imaging have been realized.^6^, ^10^ ^]^ Both graphitic shell and plasmonic core have superior TPL performance, endowing PNGs with potential for Raman and TPL dual‐modal imaging of cancer cells or tissues.^[^ ^7^, ^11^ ^]^ Based on the unique magnetic properties of MGNs, MRI of pH,^[^ ^12^ ^]^ in situ targeted MRI detection of *Helicobacter pylori* ^[^ ^13^ ^]^ and imaging‐guided, magnetically targeted gastric oral drug delivery^[^ ^14^ ^]^ have been realized.

Third, metal graphitic nanocapsules have also been used for photothermal therapy (PTT), synergistic thermo‐chemotherapy, and antibacterial therapy. Due to the superior near infrared (NIR) light absorption capacity of plasmonic core and graphitic shell, PGNs have been used for NIR laser mediated PTT of pathogenic bacteria, cancer cells, and solid tumors.^[^ ^15^ ^]^ The graphitic shell with large specific surface area and delocalized π electronic structure can be robust platform for chemotherapeutic drugs loading, PGNs have been used for synergistic thermo‐chemotherapy of cancer cells.^[^ ^7^ ^]^ In addition, the multifunctional ELGNs with multienzyme‐like activity, magnetism, and NIR light absorption capacity have been used for ablation of solid tumors^[^ ^16^ ^]^ and selective killing of *H. pylori* in vivo.^[^ ^17^ ^]^

In this review, we first introduce the methods for metal graphitic nanocapsules preparation, the specific mechanism of CVD growth process is also discussed. Subsequently, properties of different types of metal graphitic nanocapsules are systematically summarized, and then the latest developments of their applications in biodetection, bioimaging, and in vitro/in vivo therapy are introduced in detail. Challenges and future prospects of metal graphitic nanocapsules in biomedicine are finally highlighted. We expect this review will enable readers to gain an in‐depth insight into the development status of the versatile metal graphitic nanocapsules in biomedicine. Meanwhile, we look forward the readers to put forward some constructive suggestions to better promote their clinical applications.

## 2 PREPARATION AND PROPERTIES OF METAL GRAPHITIC NANOCAPSULES

### 2.1 Preparation and mechanism

Various synthesis methods for metal graphitic nanocapsules have been reported, including radio frequency thermal plasma,^[^ ^18^ ^]^ arc‐discharge,^[^ ^19^ ^]^ wet chemical synthesis,^[^ ^20^ ^]^ and CVD methods.^3^, ^4^, ^5^ ^]^ It is very difficult to obtain controllable size and shape as well as high‐quality metal graphitic nanocapsules by the arc‐discharge method. Mass production of metal graphitic nanocapsules can be realized by the wet chemical synthesis method, but the harsh reaction condition makes it difficult to prepare graphitic materials with designed functions. In comparison, the CVD method is easier to control and can be used for the growth of large‐area high‐quality graphene, conferring advantages in metal graphitic nanocapsules preparation.^3^, ^4^, ^5^ ^]^ This section mainly discusses the CVD growth process and mechanism for metal graphitic nanocapsules preparation, and the properties are also introduced in detail.

**FIGURE 1:**
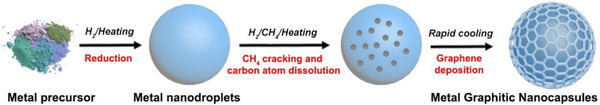
Procedure and mechanism of fumed SiO_2_ template mediated CVD method for metal graphitic nanocapsules synthesis

The mechanism for metal graphitic nanocapsules preparation is similar to that of the traditional bulk metal‐catalyzed graphene CVD synthesis.^[^ ^21^ ^]^ The metal substrate acts as the catalyst to reduce the reaction energy barrier, and meanwhile, the direct diffusion or dissolution and segregation of carbon atoms on its surface determine the graphene's quality and thickness.^21^, ^22^ ^]^ Generally, direct diffusion or dissolution and segregation of carbon atoms coexist in the graphene CVD processes, but which route dominates rely on the metal substrate's intrinsic properties.^[^ ^23^ ^]^ Taking the fumed SiO_2_ template mediated CVD method for metal graphitic nanocapsules preparation as an example, the metal catalyst confined in the SiO_2_ template firstly becomes nanodroplets under reduction and high temperature conditions, then the saturated carbon atoms formed by the pyrolysis of the carbon source (mainly methane) are dissolved and deposited on the metal catalyst surface, and the graphene is finally attached on the metal catalyst surface after rapid cooling (Figure 1).^[5b^ ^]^ The collected products are etched with HF, cleaned by centrifugation and washing to obtain pure metal graphitic nanocapsules, and covalent or non‐covalent functionalization method is further used for improving their water solubility and biocompatibility. Oxidation reaction^[6c^ ^]^ and click reaction^[^ ^24^ ^]^ mediated covalent modification methods may destroy the integrity of the graphitic shell. Currently, polyoxyethylenestearyl ether (C_18_‐PEG), a biocompatible amphipathic polymer, is commonly used to functionalize metal graphitic nanocapsules via hydrophobic interactions for improving their water solubility and reducing nonspecific adsorption to biomolecules.^4^, ^5^ ^]^

### 2.2 Properties

On one hand, metal graphitic nanocapsules exhibit some inherent characteristics of graphitic nanomaterials.^4^, ^9^, ^16^, ^25^ ^]^ Transmission electron microscopy (TEM) images show they structurally consist of a metal or alloy core encapsulated in graphitic shell. Metal graphitic nanocapsules have an ultraviolet–visible (UV–vis) absorption peak at ∼265 nm from the graphitic shell, and are able to quench the fluorescence signal through fluorescence resonance energy transfer (FRET) process. Due to the protection of graphitic shell, metal graphitic nanocapsules show superior stability in acid, alkali, salt solution, and redox conditions. metal graphitic nanocapsules have a disordered (D) band at ∼1330 cm^−1^ and a graphitic carbon (G) band at ∼1590 cm^−1^ from the graphitic shell, while the intensity of the 2D peak (∼2650 cm^−1^) in the Raman silent region (1800–2800 cm^−1^) is mainly determined by the thickness of the graphitic shell. The characteristic Raman bands themselves can be used as Raman signal labels or internal standard (IS) to improve the reliability of Raman analysis. Benefiting from the large specific surface area and delocalized π electronic structure of the graphitic shell, drug and targeting molecules can be loaded on their surface via hydrophobic or π–π interactions, which further broadens their applications in biomedicine.

On the other hand, the specific properties of various metal graphite nanocapsules depend on their composition, size, and morphology of the metal cores, and further affect their biomedical applications.^4^, ^9^, ^16^, ^25^ ^]^ According to the composition and properties of the metal core, they can be divided into four types, including plasma PGNs, MGNs, MPGNs, and ELGNs. The metal cores of PGNs include Au, Ag, Cu, and their alloys.^6^, ^7^, ^11^, ^26^ ^]^ Optical properties of the internal metal core determine the UV–vis absorption spectrum characteristics of PGNs. Benefiting from their excellent plasmonic activity, PGNs have been widely used for SERS detection and imaging,^6^, ^26^, ^27^ ^]^ TPL imaging,^[^ ^11^ ^]^ and light‐mediated therapy.^[^ ^15^ ^]^ The metal cores of MGNs include Co and FeCo.^[^ ^8^, ^13^, ^14^ ^]^ Due to the excellent magnetic properties of MGNs, magnetic separation and enrichment of biological samples for improved detection sensitivity,^[8a^ ^]^ MRI of *H. pylori* in vivo^[^ ^13^ ^]^ and magnetic targeted drug delivery applications^[^ ^14^ ^]^ have been realized. MPGNs possess the characteristics of both PGNs and MGNs. Up to now, Au nanoparticles modified multilayered graphitic magnetic nanocapsules (AGNs)^[6c^ ^]^ and magnetic graphene‐isolated AuCo nanocrystals (MACGs)^[6d^ ^]^ have been reported for separation and enrichment of biological samples, as well as sensitive SERS bioanalysis and imaging. ELGNs mainly include Ru, CoRu, and CoPt alloys and their particle sizes are less than 5 nm.^[^ ^9^, ^16^, ^25^ ^]^ Ultrasmall ELGNs have large surface‐area‐to‐volume ratios and rich unsaturated sites for the adsorption of reactant species, showing superior multienzyme‐like activity. Generally, ultrasmall nanoparticles (NPs) have low adsorption energy and poor stability, but ultrasmall ELGNs have superior stability and robust enzyme‐like activities in harsh conditions, which is due to the fact that few‐layer graphitic shell effectively protects the metal core from dissolution and agglomeration, and meanwhile, their catalytic activities are not greatly affected. Based on the above outstanding merits of ELGNs, colorimetric detection,^[^ ^9^ ^]^ therapy of pathogens^[^ ^17^ ^]^ and solid tumors^[^ ^16^ ^]^ have been explored.

In summary, metal graphitic nanocapsules have the unique properties of both graphitic and metal nanomaterials. For the convenience of readers, similar and different properties of the above four types of metal graphitic nanocapsules and their applications in biomedicine are shown in Table 1.

**TABLE 1:** Properties and applications of metal graphitic nanocapsules

Types	Similar properties	Different properties	Applications
PGNs	Superior stability; high specific surface area; unique Raman scattering bands; fluorescence quenching property; easy to functionalize and assemble, good biocompatibility	Unique local surface plasmon resonance (LSPR) properties; good photothermal effect	SERS detection and imaging; TPL imaging, PTT applications
MGNs		Magnetism	MRI applications; magnetic separation and enrichment
MPGNs		Unique LSPR properties; Magnetism	SERS detection and imaging
ELGNs		Multienzyme‐like activity	Colorimetric detection, Treatment of pathogens and tumors

## 3 BIOMEDICAL APPLICATIONS OF METAL GRAPHITIC NANOCAPSULES

Novel metal graphitic nanocapsules have the properties of both graphitic and metal nanomaterials, exhibiting great potential in the field of biomedicine. Based on the superior stability, good fluorescence quenching performance, unique Raman scattering bands, large specific surface area and delocalized π electronic structure of the graphitic shell, as well as the unique optical and magnetic properties of different metal cores, a variety of functional applications have been realized. The applications of metal graphitic nanocapsules in biodetection, bioimaging, and therapy will be introduced in detail.

### 3.1 Biodetection

SERS spectroscopy technology, a robust optical technique inherits the inherent advantages of Raman spectroscopy and improves detection sensitivity through the excitation of precious metal NPs with LSPR effect, has been extensively used for bioanalysis.^[^ ^28^ ^]^ SERS bioanalysis involves the relationship between the SERS substrate and complex biological environment, which means the reasonable design of SERS substrate is of great significance.^5^, ^29^ ^]^ Due to the protection of few‐layer graphitic shell, PGNs have superior stability in harsh conditions, the direct contact between analytes and the SERS substrate is also avoided, photocarbonization of analytes and unnecessary reactions are minimized, resulting in more reliable SERS analytical results.^[^ ^30^ ^]^ The graphitic shell can quench the background fluorescence of the biosystem through the FRET process, which is beneficial to improve the reliability of SERS analysis. In addition, they also have unique Raman scattering characteristic bands, especially the 2D band in the Raman silent region can be used as a stable and non‐interference IS to calibrate the errors caused by experimental factors (including long‐term laser irradiation, difference in concentration depth, and fluctuations of SERS substrate) and detection environment (including high concentration of acid, alkali, salt, protein, etc.) mediated signal fluctuations, which effectively guarantees the accuracy of SERS quantification to a certain extent.^[^ ^5^ ^]^

**FIGURE 2:**
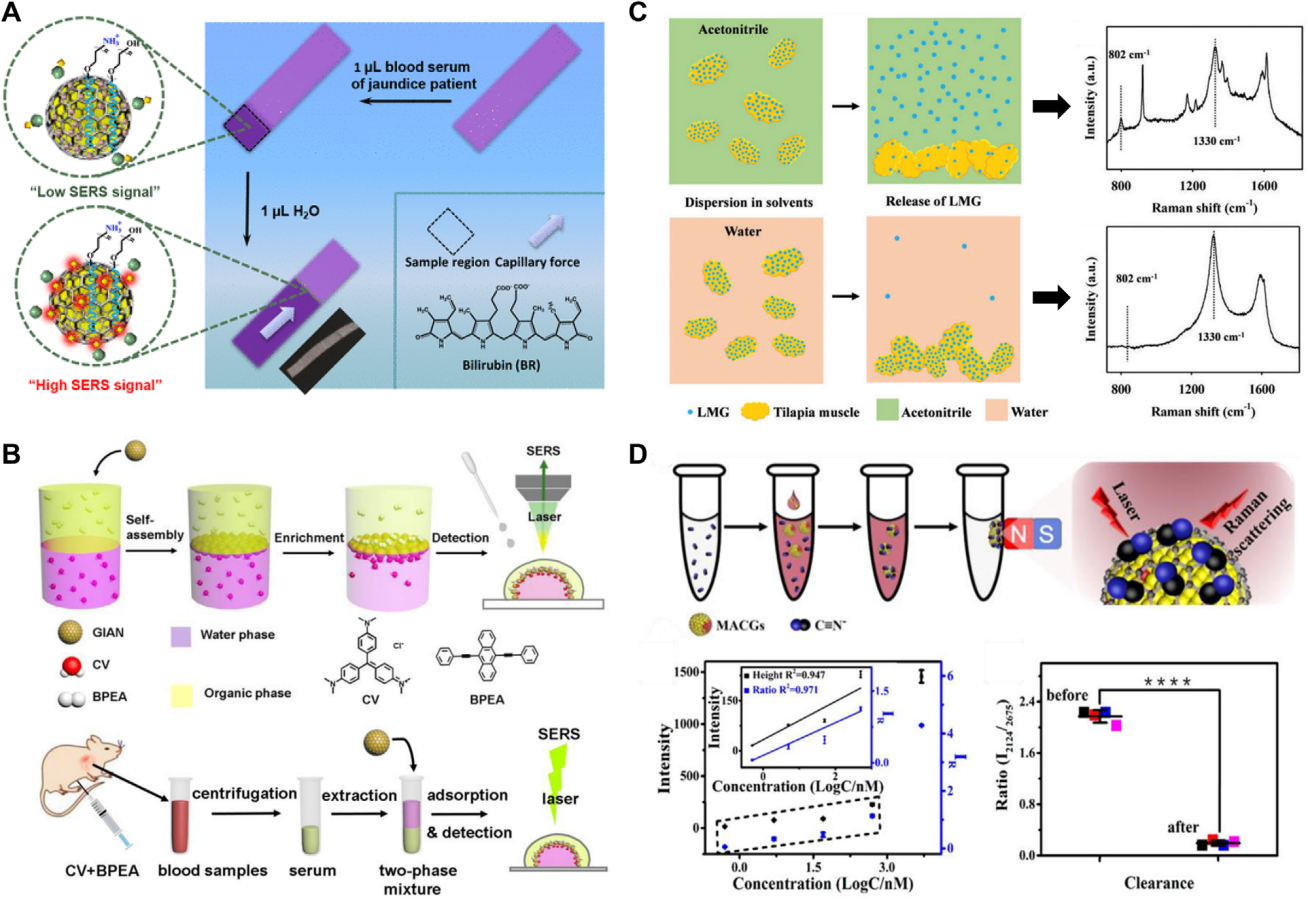
Metal graphitic nanocapsules for SERS bioanalysis. (A) The adsorption process of BR occurred on the surface of GIANs in fetal bovine serum (FBS) matrix. Insert image was the digital photo of CS@GIANs dripped with BR (FBS) aqueous after separation. Reproduced with permission.^[6b^ ^]^ Copyright 2018, American Chemical Society. (B) Schematic diagram of multiphase Raman detection of CV and BPEA with GIANs in test tube and in the blood of mice. Reproduced with permission.^[^ ^10^ ^]^ Copyright 2018, American Chemical Society. (C) SERS detection of LMG in contaminated fish muscle washed with different solvents. Reproduced with permission.^[^ ^24^ ^]^ Copyright 2018, Royal Society of Chemistry. (D) Schematic diagram of CN^−^ enrichment processes MACGs and the results for CN^−^ clearance ability of MACGs in the water sample. Reproduced with permission.^[6d^ ^]^ Copyright 2019, American Chemical Society

Bian et al.^[^ ^7^ ^]^ firstly report the application of ultra‐stable graphene‐isolated‐Au‐nanocrystals (GIANs) as the SERS substrate, an enhancement factor > 100 for the SERS analysis of rhodamine 6G and minimized photobleaching of analyte have been achieved, indicating the excellent SERS analysis performance of GIANs. Afterward, Zhang et al.^[6a^ ^]^ report the application of GIANs for quantitative SERS analysis of crystal violet (CV, a common antibacterial agent), and the 2D band in the Raman silent region is act as an IS to calibrate the deviation caused by external environments. Taking advantage of GIANs’ large surface area and superior SERS activity, Zhao et al.^[^ ^31^ ^]^ use a simple ultrasound method to prepare an alkyne functionalized GIANs SERS nanoprobe within several minutes for sensitive ratiometric detection of alkaline phosphatase with acetonitrile as the IS. Although SERS technique provides rich fingerprint information and possesses very high sensitivity,^29^, ^32^ ^]^ SERS‐based point‐of‐care (POC) tests face interference of many other complex molecules in biosamples, especially the competitive adsorption of non‐target molecules on the SERS substrate probably cause the inaccurate of analysis results.^[^ ^33^ ^]^ Taking the detection of free bilirubin (BR) related to the pathogenesis of jaundice as an example, the bilirubin is commonly combined with albumin in the serum, which brings a huge challenge to the POC detection of bilirubin based on SERS technology.^[^ ^34^ ^]^ Based on the graphitic shell of GIANs can extract BR from albumin and the separation, enrichment capacity of cellulose strips (CS), Zou et al.^[6b^ ^]^ prepare a GIANs‐loaded CS platform to realize the SERS detection of free BR in trace blood samples, and G band is used as the IS to calibrate the test results, indicating that GIANs provide a superior anti‐interference and highly sensitive SERS platform for POC tests in clinical scenarios (Figure 2A).

The above works use GIANs as the SERS substrate to achieve single‐phase bioanalysis. However, in many cases, the analytes in the actual sample are mixture dispersed in different phases, such as lipid‐ and water‐soluble drugs are often administered simultaneously for synergistic treatment.^[^ ^35^ ^]^ It is usually necessary to use appropriate solvents to dissolve multiple analytes with different solubility for realizing their Raman analysis, which seriously affects the accuracy of the analysis results. Zhang et al.^[^ ^10^ ^]^ propose a simple, sensitive, and simultaneous multiphase enrichment SERS detection strategy based on the self‐assembly of GIANs at the immiscible two‐phase interface without any surface modification and inducer. Taking the 9,10‐bis(benzene) phenylethynyl)anthracene (BPEA) and CV as the model molecules to simulate lipid‐ and water‐soluble drugs in the blood, simultaneous multiple SERS detection of these two drugs are executed by the interface self‐assembled GIANs membrane, and the 2D band is further used as the IS to calibrate the analysis results (Figure 2B). The GIANs‐based SERS analysis platform constructed in this work shows superior multiplex detection capabilities and application prospects in future clinical drug monitoring. Recently, Tang et al.^[^ ^36^ ^]^ report a laser‐mediated highly efficient analyte enrichment strategy for sensitive SERS detection of drug model molecules using the interfacial assembled GIANs as the SERS substrate and analyte enrichment platform. The proposed strategy avoids the signal fluctuation caused by the “coffee ring effect” and is expected to provide a reliable platform for SERS analysis of complex samples.

Compared with Au nanocrystals (Au NCs), Ag nanocrystals (Ag NCs) have larger optical cross section and more acceptable price, but Ag NCs are easily oxidized, which hinders their SERS analysis applications.^[^ ^37^ ^]^ Like GIANs, confining the Ag NPs within inert graphitic shell via CVD method can effectively protect the Ag core from the interference of external environment. However, CVD method‐based graphene‐isolated Ag NCs have rarely been reported since the poor catalytic activity of Ag makes it difficult to grow graphene on its surface. Herein, Song et al.^[26b^ ^]^ report a graphene‐encapsulated AgCu nanocrystal (AgCu@G) using CVD method to achieve the growth of few‐layer graphene on Ag surface by taking advantage of the superior catalytic properties of Cu, and significantly enhanced Raman signal of rhodamine 6G has been obtained. Subsequently, Song et al.^[^ ^24^ ^]^ further use the amphiphilic molecules modified AgCu@G as the SERS substrate to realize the detection of water‐soluble malachite green (MG) and lipid‐soluble leuco malachite green (LMG) in tilapia muscle samples, and the D band is used as the IS to calibrate the detection results (Figure 2C). Recently, Li et al.^[26c^ ^]^ report another Ag NCs‐contained PGNs of graphene‐isolated AuAg nanocrystal (GIAAN) for sensitive SERS detection of CV and MG in homogeneous systems. The above works indicate CVD method offers an alternative strategy to prepare stable Ag NCs‐based substrates for sensitive SERS bioanalysis.

MPGNs have the unique property of both PGNs and MGNs, the superior magnetic separation and enrichment properties endow them with huge potential for improving detection sensitivity of SERS bioanalysis. Zou et al.^[6c^ ^]^ report the first MPGN of AGNs, which structurally show large amount of Au NPs modified on multilayered graphitic magnetic nanocapsules surface. Strong electromagnetic hot spot gaps generated among Au NPs greatly enhance the Raman signal of rhodamine B, the 2D band is used as the IS to reduce the heterogeneity of the substrate surface, and the detection limit is as low as 40 fmol. However, the AGNs ignore the stability of the plasmonic Au NPs, which makes it very important to prepare novel MPGN with an alloy core containing both magnetic and plasma active metal nanomaterials encapsulated in a graphite shell. Subsequently, Zhang et al.^[6d^ ^]^ report another MPGN of ultra‐stable magnetic graphene‐isolated AuCo nanocrystal (MACGs), exhibiting superior magnetic separation and enrichment capabilities as well as superior corrosion resistance performance. Significantly, although graphene isolates the direct contact between Au and CN^−^, their interaction is transferable and remains, which gifts MACGs superior direct CN^−^ capture capability. Based on that, efficient CN^−^ capture and clearance in various hydrologic environments have been realized, and the 2D peak is used as the IS to improve the accuracy of SERS quantification (Figure 2D).

**FIGURE 3:**
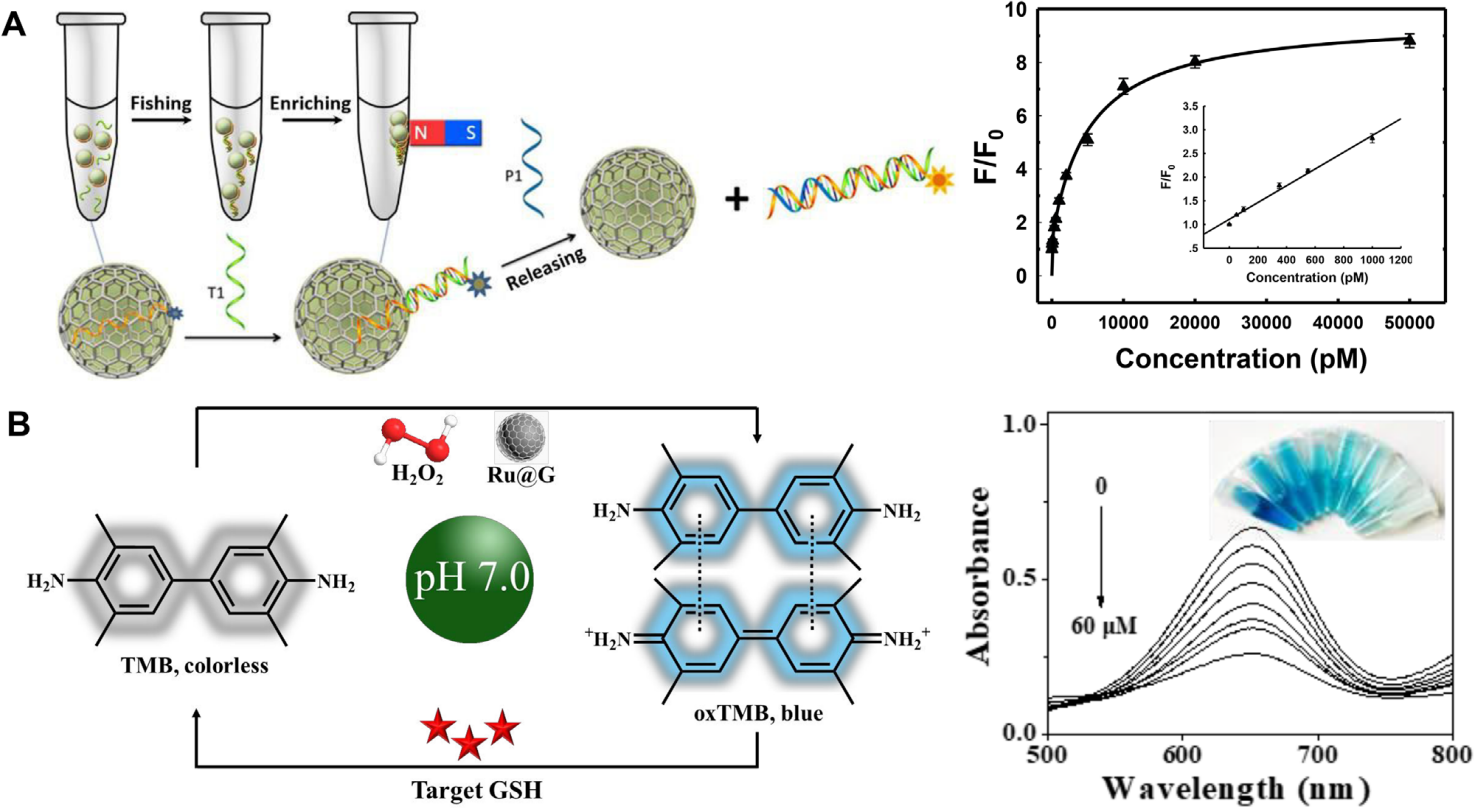
Metal graphitic nanocapsules for fluorescence and colorimetric bioanalysis. (A) Schematic diagram of Co@G for fluorescence detection of DNA and the corresponding experimental results. Reproduced with permission.^[8a^ ^]^ Copyright 2013, Wiley‐VCH. (B) Principle for colorimetric detection of GSH and corresponding experimental results. Reproduced with permission.^[^ ^9^ ^]^ Copyright 2021, Royal Society of Chemistry

Due to the unique magnetic properties of the metal core and superior fluorescence quenching performance of the graphitic shell, some MGNs‐based fluorescence detection platforms have been reported. Song et al.^[8a^ ^]^ report a Co‐encapsulated graphitic nanocapsules (Co@G)‐based efficient DNA capture and release strategy to achieve highly sensitive fluorescence detection of DNA, and threefold lower limit of detection has been obtained compared with carbon nanotube and graphene‐based fluorescent biosensors (Figure 3A). Han et al.^[8b^ ^]^ further use Co@G to achieve sensitive fluorescence detection of histone acetyltransferase activity, and they find its fluorescence quenching performance is superior to that of graphene oxide, single‐walled carbon nanotubes, and Au NPs. In addition, Nie et al.^[^ ^12^ ^]^ use FeCo‐encapsulated graphitic nanocapsule (FeCo@G) to load photoactive molecules for fluorescence‐ and MRI relaxivity‐based pH monitoring, indicating the potential of FeCo@G as a pH‐responsive MRI contrast agent for stomach diseases diagnosis. These works prove MGNs can be powerful tools for molecular disease diagnosis.

Nanozymes show lower catalytic activities than natural enzymes, which limit their extensive applications in bioanalysis.^[^ ^38^ ^]^ Enhanced enzyme‐like activity can be obtained by reducing the particle size because smaller NPs have larger surface area‐to‐volume ratios and more unsaturated sites for reactant species adsorption.^[^ ^25^, ^39^ ^]^ However, ultrasmall NPs have low adsorption energy and poor stability, which makes it difficult to prepare superstable and ultrasmall nanozymes. CVD method prepared ELGNs in which few‐layer graphitic shell protects the ultrasmall‐sized catalytically active metal NPs from dissolution and agglomeration, showing great promise in bioanalysis. Recently, Keoingthong et al.^[^ ^9^ ^]^ report that the Ru encapsulated graphitic nanocrystal (Ru@G) exhibits superior peroxidase‐like activity over a broad pH range and in different harsh conditions. In the presence of H_2_O_2_, the peroxidase‐like Ru@G catalyze the oxidation of colorless 3,3′,5,5′‐tetramethylbenzidine (TMB) to blue‐colored oxidized TMB (oxTMB) with a characteristic UV–vis absorption peak at 652 nm, while the formation of oxTMB is inhibited with the addition of glutathione (GSH). Therefore, sensitive colorimetric detection of GSH at near‐physiological pH has been eventually realized (Figure 3B).

### 3.2 Bioimaging applications

Optical imaging techniques have some inherent advantages like high sensitivity, specificity and resolution can provide more accurate multi‐parameter information for bioanalysis, showing great promise in bioimaging.^[^ ^40^ ^]^ Among the commonly used optical imaging techniques, Raman imaging technique, a molecular imaging technique with high spatial resolution, photostability, and multiplexing capabilities, has been widely used to characterize some important biological processes at the cellular and subcellular level, especially showing significant advantages in the field of imaging‐mediated therapy.^29^, ^41^ ^]^ Metal graphitic nanocapsules have larger Raman scattering cross section and much better stability than traditional organic Raman tags, endowing them with potential for multimodal cellular Raman imaging, especially the 2D band located in the cellular silent region as imaging tag can effectively avoid the interference from complex physiological environment.^[4c^ ^]^ In addition, LSPR effects of the metal cores endow PGNs with superior TPL imaging capability, Raman and TPL dual‐modal imaging of cancer cells or tissues have been therefore realized.^[^ ^7^ ^]^

**FIGURE 4:**
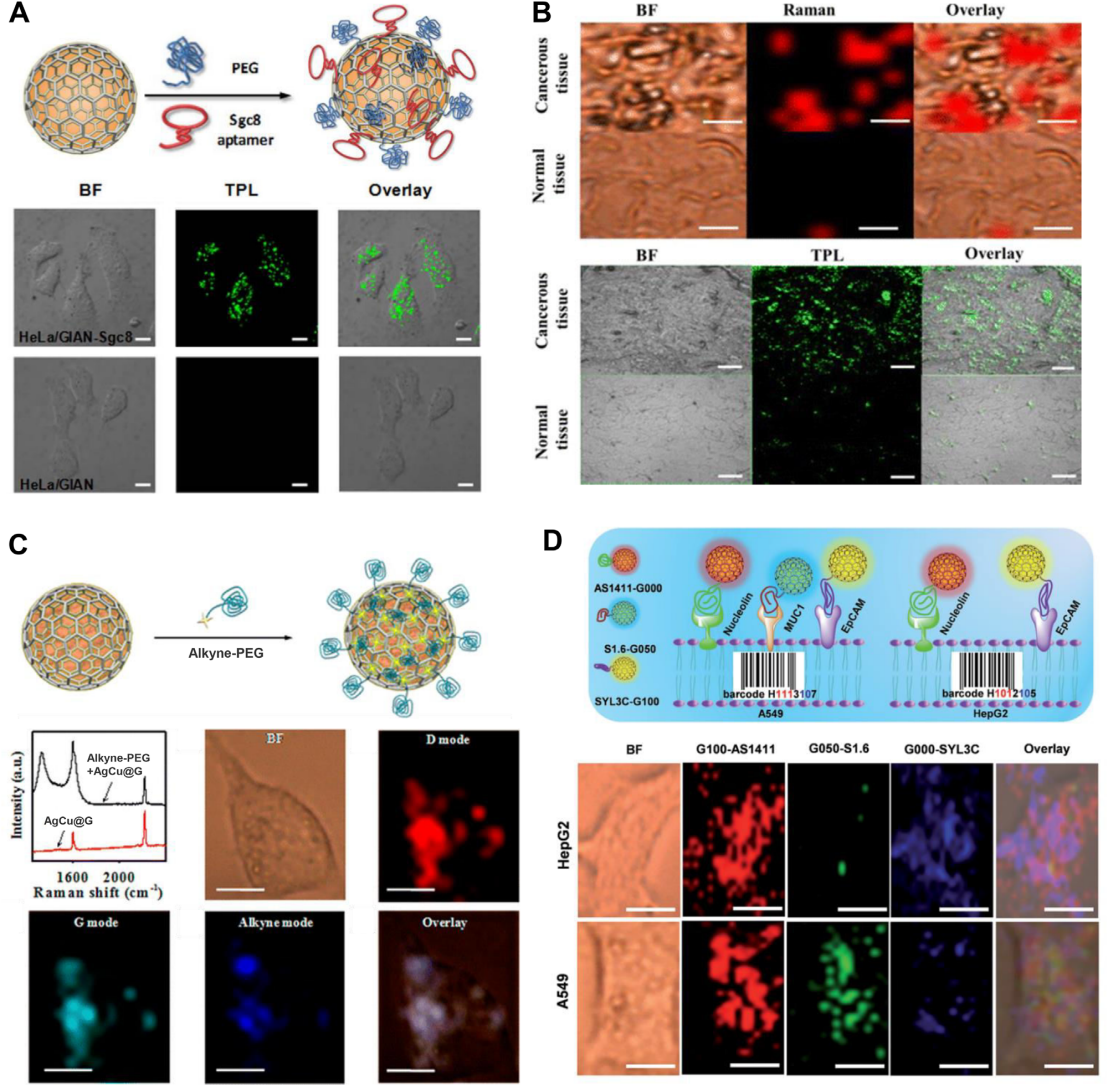
Metal graphitic nanocapsules for Raman and TPL imaging. (A) Schematic illustration of Sgc8 aptamer‐functionalized GIAN and TPL confocal images of HeLa incubated with GIAN and GIAN‐Sgc8. BF: bright field, scale bar: 10 mm. Reproduced with permission.^[^ ^7^ ^]^ Copyright 2014, Nature Publishing Group. (B) TPL confocal images of HeLa incubated with GIAN and GIAN‐Sgc8. BF: bright field, scale bar: 10 mm. Reproduced with permission.^[^ ^11^ ^]^ Copyright 2016, American Chemical Society. (C) Schematic illustration of the alkyne‐PEG functionalization of AgCu@G, Raman spectra of alkyne‐PEG with (black) and without (red) AgCu@G and Raman image of MCF‐7 cells treated with alkyne‐PEG‐modified AgCu@G BF, bright field; scale bar, 10 μm. Reproduced with permission.^[26b^ ^]^ Copyright 2014, American Chemical Society. (D) SERS images of cancer cells, scale bar, 10 mm. G100, G050, and G000 conjugated with DSPE‐PEG‐linked aptamer AS1411, S1.6, and SYL3C, respectively. Reproduced with permission.^[^ ^27^ ^]^ Copyright 2018, Royal Society of Chemistry

Bian et al.^[^ ^7^ ^]^ report the use of the GIANs’ D and G‐bands as signal labels for Raman imaging of human breast cancer cells MCF‐7, and further use the TPL performance of the Au core and graphitic shell for TPL imaging of MCF‐7 cells, the Raman and TPL dual‐modal imaging method improves the accuracy of intracellular co‐localization ability of GIANs. Besides, the Sgc‐8 aptamer that can specifically recognize protein tyrosine kinase 7 (PTK7)^[^ ^42^ ^]^ is modified on GIANs’ surface via π–π interaction to obtain Sgc‐8 functionalized GIANs, Hela cells with high PTK‐7 expression and 95‐C lung cancer cells with low PTK‐7 expression has been finally distinguished, proving GIANs have excellent cellular targeted imaging capabilities (Figure 4A). Subsequently, Wang et al.^[^ ^43^ ^]^ further study the difference between GIANs and Sgc‐8 aptamer‐functionalized GIANs entering Hela cells by Raman and TPL dual‐modal imaging. The experimental results show that nonfunctionalized GIANs entered the HeLa cells via a caveolae‐mediated endocytosis pathway, whereas functionalized GIANs are taken up by the cells via a clathrin‐mediated endocytosis process during which the NPs firstly bind to a cell surface receptor, followed by internalization of the NPs through invagination of the cell membrane. This work offers a good platform to promote a better understanding of NPs endocytosis and metabolism in cells for reliable biomedical applications.

Anisotropic gold nanorods (AuNRs) have larger optical absorption cross‐sections than Au NPs.^[^ ^44^ ^]^ Lai et al.^[^ ^11^ ^]^ use a confined CVD method to prepare AuNR‐encapsulated graphitic nanocapsules (AuNR@G) for multimodal Raman and TPL imaging of MCF‐7 cells. As compared, Au NP‐encapsulated graphitic nanocapsules (Au@G) by the similar confined CVD method is also prepared, and the experimental results indicate the AuNR@G shows significantly better Raman and TPL cellular imaging performance than that of Au@G. The SYL3C aptamer that can specifically target epithelial cell adhesion molecules^[^ ^45^ ^]^ is further immobilized on AuNR@G surface for the recognition of cancer and normal tissues through Raman and TPL dual‐modal imaging, proving AuNR@G has excellent tissue‐targeted imaging capabilities (Figure 4B). In addition, based on the MACGs can direct capture CN^−^, Zhang et al. establish a *Pseudomonas aeruginosa*‐infected *Caenorhabditis elegans* model, D and G modal Raman imaging firstly prove MACGs enter the body of *C. elegans*, sensitive SERS imaging detection of the CN^−^ (an important biomarker of *Pseudomonas aeruginosa*)^[^ ^46^ ^]^ in vivo has been further realized.

The D and G bands overlap with the inherent signals within the cell, which limits the sensitivity and accuracy of SERS imaging to a certain extent. Using the Raman reporter molecules in the cellular silent region as the signal probe can improve the accuracy of Raman imaging, which is one of the research hotspots in the field of Raman bioimaging.^[^ ^47^ ^]^ Song et al.^[26b^ ^]^ use the D, G bands and the alkynyl group located at ∼2220 cm^−1^ of alkynyl‐PEG functionalized AgCu@G for multi‐modal Raman imaging of MCF‐7 cells, and the introduction of alkynyl‐PEG improves the accuracy of Raman imaging for monitoring endocytosis processes of AgCu@G inside cells. Alkynyl‐PEG functionalized AgCu@G Raman tags are further immobilized with specific aptamers for targeted cell and tissue Raman imaging, showing great potential in clinical applications (Figure 4C). However, the cumbersome procedure for alkynyl‐PEG functionalization and poor stability of alkynyl‐PEG in harsh environments probably hinder the extensive application of AgCu@G in Raman imaging. The stable 2D band of GIAN in the cellular silent region as an imaging probe can solve the above problems to a certain extent. Based on this, Zou et al.^[^ ^27^ ^]^ prepare five GIANs with different 2D band shifts as SERS imaging tags by changing the ratio of isotope ^12^C and ^13^C in the CVD system. Background‐free SERS imaging of A549 cells and *C. elegans* firstly prove the multiplexed imaging capabilities of GIANs in vitro and in vivo. Furthermore, aptamers that can specifically recognize membrane proteins are modified on the surface of the SERS‐encoded GIANs tag as a built‐in code for fast imaging and pattern recognition of HepG2 and A549 cell lines. The isotope GIAN‐aptamer encoders have great potential in highly sensitive cancer cell identification free from background interference, thus providing a powerful and valuable platform for future clinical disease diagnosis (Figure 4D).

**FIGURE 5:**
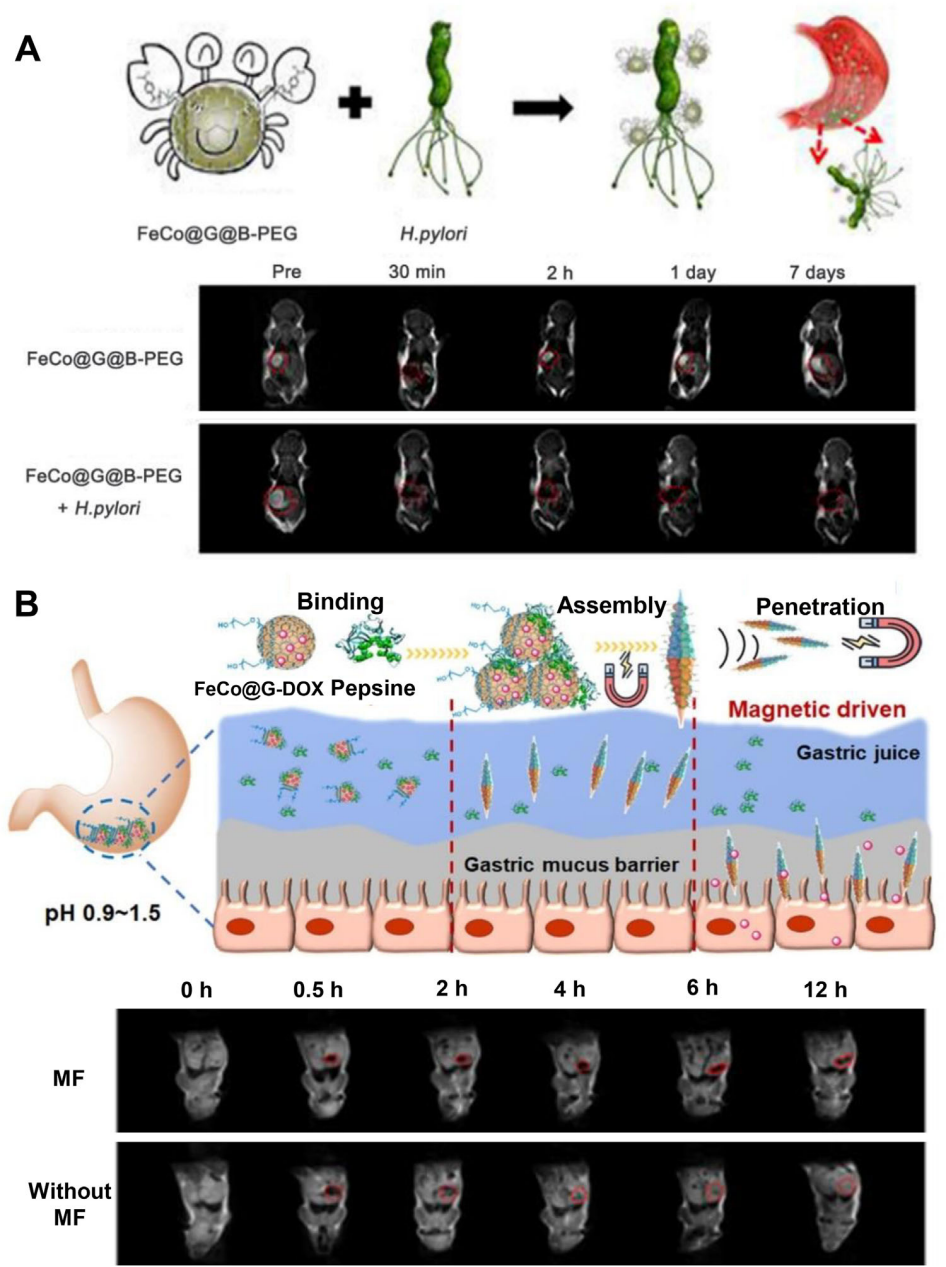
Metal graphitic nanocapsules for MRI applications. (A) Schematic illustration of *H. pylori* detection with FeCo@G@B‐PEG and *T*_2_‐weighted images at different times with or without FeCo@G@B‐PEG treatments. Reproduced with permission.^[^ ^13^ ^]^ Copyright 2017, Nature Publishing Group. (B) Illustration of gastric retention and penetration in vivo and *T*_2_‐weighted MRI of BALB/c mice after administration with FeCo@G@B‐PEG at different times with and without application of magnetic field (MF). Reproduced with permission.^[^ ^14^ ^]^ Copyright 2021, Elsevier

The penetration depth of Raman and TPL imaging techniques is limited, making them difficult to achieve in vivo imaging diagnostic applications of mammals. The MRI technology, a novel technique developed in recent decades based on the interaction between protons and the surrounding molecules of tissues, playing a vital role in molecular imaging and clinical diagnosis due to its noninvasiveness, high spatial and temporal resolution, and high penetration depth.^47^, ^48^ ^]^ MRI offers detailed information on abnormal anatomy in a 3D tomographic and real‐time manner with soft tissue contrast.^48^, ^49^ ^]^ MRI uses contrast agents such as Gd^3+^, Mn^2+^, and superparamagnetic iron oxide NPs to enhance the signal,^[^ ^50^ ^]^ but these commonly used contrast agents are unstable in extremely acidic environment, and even cause obvious toxic side effects or affect the proton relaxation between the magnetic nucleus and the surrounding environment, making it difficult to diagnose gastric diseases. MGNs have superior magnetic properties and excellent acid stability holding huge potential in gastric MRI applications. Li et al.^[^ ^13^ ^]^ report a phenylboronic acid‐PEG (B‐PEG) modified FeCo@G system for in situ targeted MRI imaging detection of *H. pylori* (a gram‐negative spiral microaerophilic bacteria that colonize garstric mucosa of rodents and major etiological factor of many gastric diseases) in mice, in which phenylboronic acid can specifically bind to peptidoglycan in bacterial cell.^[^ ^51^ ^]^ The *H. pylori*‐infected mice are finally sacrificed and obvious Raman signals of D and G bands are observed on the Raman imaging of stomach tissues, which further verify the FeCo@G offered a robust platform for *H. pylori* targeted imaging detection in vivo (Figure 5A).

Strong acidic and protease‐rich stomach environment, obstruction of the mucus barrier, and short residence time of oral drugs severely hinder the effective delivery of oral medications in the stomach.^[^ ^52^ ^]^ Progress has been made in magnetically targeted drug delivery, especially the controlled assembly of magnetic nanomaterials can contribute to the targeted delivery of drug molecules.^[^ ^53^ ^]^ However, the instability of magnetic drug‐loading platform in the harsh gastric environment makes it difficult to achieve in situ controllable assembly, which greatly limits the efficiency of drug delivery. Luckily, MGNs show superior magnetic properties and excellent stability in harsh gastric environment. Cai et al.^[^ ^14^ ^]^ develop a synergistically pepsin‐bridged and magnetic field‐mediated FeCo@G needle‐like assembly, showing better cell membrane penetration ability and magnetic response movement ability in simulated gastric milieu than that of FeCo@G. Model antitumor drug of doxorubicin (DOX) loaded FeCo@G needle‐like assembly also has better gastric cancer cell killing performance than DOX loaded FeCo@G. Based on that, the inherent pepsin in the stomach environment is further used as a “bridge” to realize the in situ assembly of FeCo@G needle‐like assembly under the mediation of an external magnetic field, MRI and Raman imaging verify that the needle‐shaped assembly can overcome the gastric mucus barrier, enhance the mucosal penetration depth and prolong drug retention time. Under imaging techniques’ guidance, we believe this magnetic field‐mediated in situ self‐assembly platform provides new ideas for the delivery of oral drugs and site‐selective treatment of gastric diseases (Figure 5B).

### 3.3 Therapy applications

In the emerging field of nanomedicine, PTT^[^ ^54^ ^]^ and thermo‐chemotherapy^[^ ^55^ ^]^ strategies have been widely used for disease treatment and pathogenic bacteria killing. During the PTT process, the photothermal agents absorb light and convert the optical energy into thermal energy to produce heat and finally induce the death of cancer cells.^[^ ^56^ ^]^ While in the thermo‐chemotherapy process, cancer cells are killed by hyperthermia and released chemotherapy drugs under the irradiation of light.^[^ ^57^ ^]^ NIR laser radiation, as a non‐invasive external stimulus, usually produces weak absorption when irradiating the skin and tissues, which effectively reduces the phototoxicity during the treatment.^[^ ^58^ ^]^ Ultra‐stable GIANs and AuNR@G with superior LSPR effect are able to effectively convert NIR light into heat through a photothermal process, and their graphitic shells also have superior NIR light absorption capabilities, conferring advantages in NIR light‐mediated PTT applications.^[^ ^7^, ^15^ ^]^ In addition, the graphitic shells of metal graphitic nanocapsules can also be used as drug or targeted molecules carrier, synergistic thermo‐chemotherapy has been achieved.^[^ ^7^ ^]^

Bian et al.^[^ ^7^ ^]^ use GIANs with good NIR light absorption properties to kill MCF‐7 cells efficiently, proving that GIANs can be used as a good photothermal reagent for cancer cells treatment. To further improve the therapeutic effect, antitumor drug DOX is further immobilized on GIANs surface to prepare the GIANs/DOX complex through π–π stacking interactions for effectively synergistic thermo‐chemotherapy of cancer cells. Under the irradiation of NIR laser, the death rate of GIAN/DOX complex‐incubated cells are much higher than that of DOX‐incubated cells, indicting the photothermal effect of GIANs effectively enhance the chemotherapy effect of individual DOX. This GIANs‐based synergistic thermo‐chemotherapy strategy not only improves the treatment efficiency, but also realizes the controllable release of DOX through the photothermal effect of GIANs to reduce the side effects of chemotherapy drugs.

**FIGURE 6:**
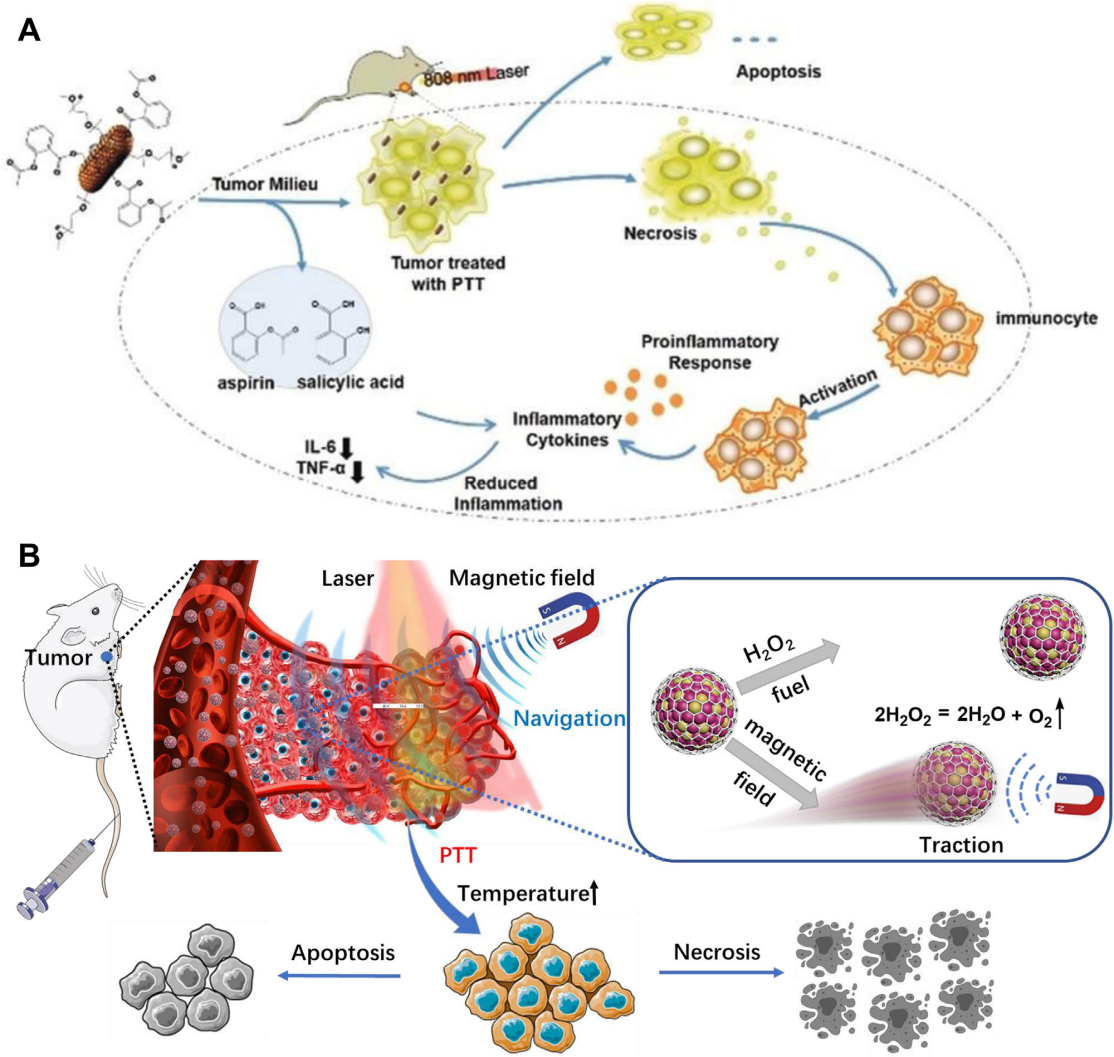
Metal graphitic nanocapsules for NIR laser mediated in vivo PTT applications. (A) Illustration of the anti‐inflammatory mechanism underlying the inhibition of PTT‐associated inflammation by AuNR@G‐P‐aspirin complex. Reproduced with permission.^[15b^ ^]^ Copyright 2018, Wiley‐VCH. (B) Illustration of the MCGNs propelled navigation to enhance intracellular delivery and PTT action against tumor cells. Reproduced with permission.^[^ ^16^ ^]^ Copyright 2021, Chinese Chemical Society

PTT avoids the side effects of chemotherapy to a certain extent, and is expected to become an alternative or supplement to traditional cancer treatments. However, excessive temperature in the PTT process damages the integrity of cell membranes and release intracellular constitutes, which further triggers inflammatory response.^[^ ^59^ ^]^ Evidence has shown that the inflammatory responses are able to cause trauma, tissue injury, and even stimulate tumor regeneration and resistance to therapy,^[^ ^60^ ^]^ making it necessary to construct a reliable PTT‐based nanoplatform to resist inflammation. Salicylate, metabolites of non‐steroidal anti‐inflammatory drug aspirin, can inhibit the production of various pro‐inflammatory cytokines, but the lack of specific drug targets and individual differences hinder the use of free pharmaceutical preparations.^[^ ^61^ ^]^ Based on this, Dong et al.^[15b^ ^]^ firstly synthesizes pyrene‐aspirin (P‐aspirin) prodrug, and then it is loaded on AuNR@G surface through hydrophobic and π–π stacking interactions to obtain AuNR@G‐P‐aspirin complex for eliminating the produced inflammatory during PTT process. The results show that the P‐aspirin prodrug is effectively released from AuNR@G‐P‐aspirin complex in the tumor microenvironment, and the levels of pro‐inflammatory cytokines return back to normal. After the AuNR@G‐P‐aspirin complex is injected in mice through the tail vein, the solid tumors are effectively ablated under the irradiation of NIR laser, and meanwhile, interleukin‐6 and tumor necrosis factor‐α are also inhibited. In addition, the AuNR@G‐P‐aspirin complex shows no obvious damage to cells around the tumor and major organs, indicating its superior biocompatibility. This work makes full use of the excellent PTT effect of AuNR@G to achieve high‐efficiency photothermal ablation of solid tumors, and meanwhile, it is used as a drug delivery platform to achieve the effective delivery of anti‐inflammatory prodrugs and eliminate the inflammatory response produced in the PTT process (Figure 6A).

Despite great progress has made in PTT of tumor, complex biological environments, and multiple physiological barriers severely impede efficient accumulation and penetration of photothermal reagents within tumor tissue for therapy.^[^ ^62^ ^]^ In situ energy conversion of nanomotors features autonomous movements and improves treatment efficiency,^[^ ^63^ ^]^ but it is very difficult to prepare nanomotors with small size, biocompatibility, and precise positioning ability. Zhang et al.^[^ ^16^ ^]^ propose a magnetocatalytic CoPt@graphene (CoPt@G) navigator (abbreviated as MCGNs) for enhanced solid tumor penetration and improved PTT efficacy (Figure 6B). First, the magnetic MCGNs act as highly diffusive delivery vehicles to promote cancer cell targeting under the navigation of an external field. Second, the ELGNs of CoPt@G with superior catalase‐like activity facilitates to produce O_2_ propelling force in the presence of H_2_O_2_ for increased cell membrane penetration depth and intracellular delivery efficiency. Third, ultrasmall MCGNs with stable MRI, photothermal, and Raman signals facilitate to diffusion among tumor cells and execute multi‐modal imaging for guiding subsequent PTT. Hence, MCGNs‐based magnetocatalytic propelling synergistic PTT of solid tumor in 4T1 tumor‐bearing mice model has been eventually achieved, and negligible biotoxicity is observed on mice. This work offers promising inspiration and opportunities for advancing cancer diagnosis and treatments. Subsequently, Xu et al.^[^ ^64^ ^]^ use the oxidase‐like CoPt@G to prepare TMB‐TMB dication (TMB^++^) complex, and an H‐aggregation of TMB‐TMB^++^ complex in linear agarose (H‐TTC/LAG) with narrowed band gap is further obtained through intermolecular hydrogen bonding between the –NH_2_ of TTC and the –NH_2_ of LAG. The H‐TTC/LAG exhibits excellent NIR‐II photothermal conversion capability and thermal stability, resulting in highly effective tumor growth inhibition in mouse mammary carcinoma. This innovative work broadens the application of ELGNs in the field of PTT.

**FIGURE 7:**
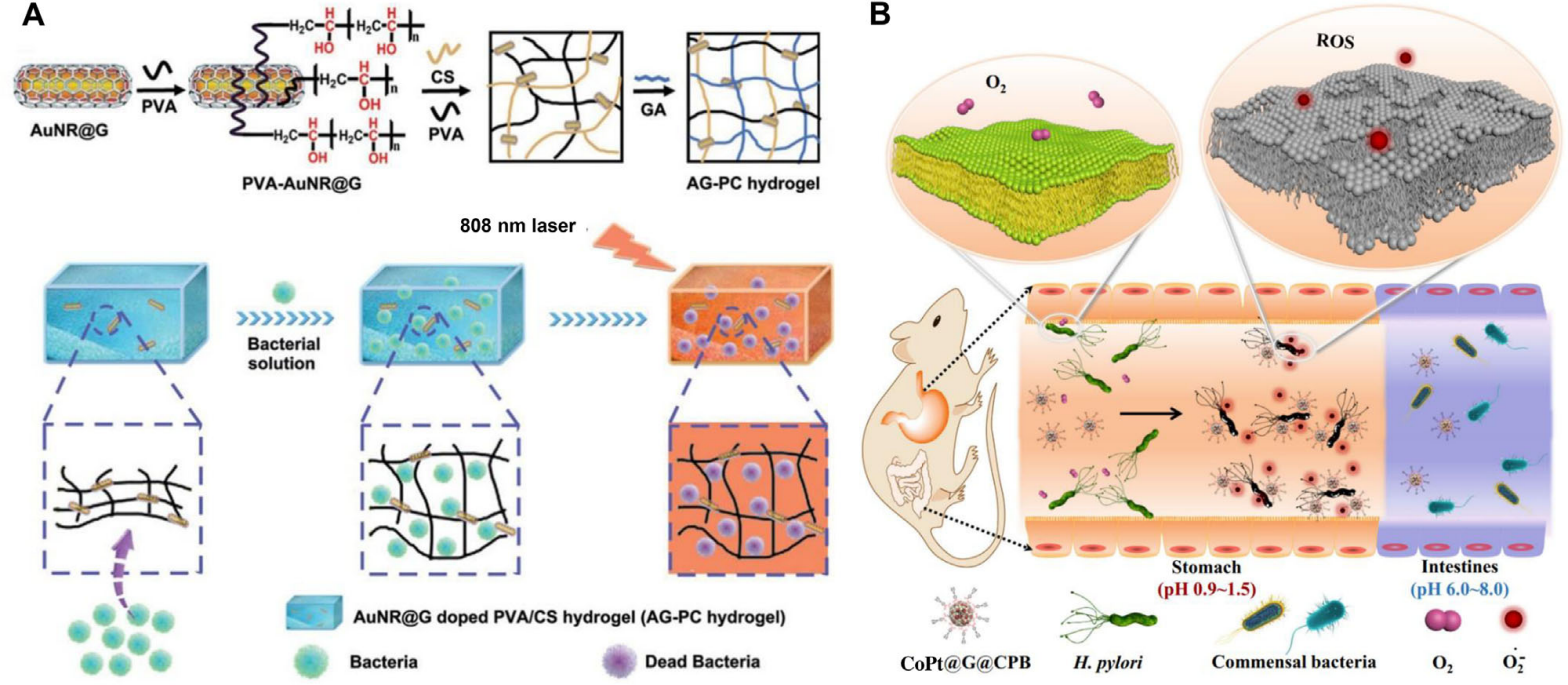
Metal graphitic nanocapsules for antibacterial applications. (A) Preparation of the AG‐PC hydrogel and NIR laser‐induced antibacterial experiments using the AG‐PC hydrogel. Reproduced with permission.^[15a^ ^]^ Copyright 2019, Royal Society of Chemistry. (B) Schematic of selective killing of *H. pylori* in vivo based on CoPt@G@CPB nanozyme. Reproduced with permission.^[^ ^64^ ^]^ Copyright 2021, Nature Publishing Group

Apart from tumor therapy, metal graphitic nanocapsules can also be used for NIR laser‐induced antibacterial treatment. Traditional antibiotic therapy cures diseases by interfering with the normal metabolic process of pathogenic bacteria, but the abuse of antibiotics inevitably increases the production of acquired drug‐resistant bacteria.^[^ ^65^ ^]^ Recent studies showed that NIR laser‐induced PTT can solve the problem of bacterial resistance to a certain degree.^[^ ^66^ ^]^ Currently, photothermal reagents are usually combined with biocompatible, flexible, and environmentally responsive hydrogels for antibacterial applications,^[^ ^67^ ^]^ but the preparation of highly stable antibiotic‐free photothermal hydrogel with minimized biosecurity is challenged. Xu et al.^[15a^ ^]^ report an AuNR@G‐doped polyethylene alcohol (PVA)/chitosan (CS) hydrogel (abbreviated as AG‐PC hydrogel) system for highly efficient PTT of both gram‐negative *Escherichia coli* and positive *Staphylococcus aureus*. Significantly, the antibiotic‐free AG‐PC hydrogel has superior physiological stability and photothermal stability, which endows it with possibility to minimize biological safety problems caused by the released photothermal reagents entering the blood circulation. We believe the AG‐PC hydrogel had broad prospects in photothermal antibacterial applications (Figure 7A).

PTT strategy has difficult in combating *H. pylori* infection in the stomach due to its limited penetration depth. Currently, triple therapy (a proton pump inhibitor and two antibiotics) is used as the standard first‐line therapy in the clinical treatment of *H. pylori* infection.^[^ ^68^ ^]^ However, the short retention time and rapid degradation of antibiotics in gastric environment severely affect the antimicrobial activity of antibiotics.^[^ ^69^ ^]^ Moreover, the abuse of antibiotics inevitably increases the risk of drug‐resistant bacteria and even affects the survival of symbiotic bacteria and further causes severe diseases.^[^ ^70^ ^]^ Therefore, it is necessary to develop an alternative strategy to achieve efficient and selective killing of *H. pylori* without affecting the healthy balance of symbiotic bacteria. Herein, Zhang et al.^[^ ^64^ ^]^ fabricate a bacteria‐targeting molecule C_18_‐PEGn‐benzeneboronic acid (CoPt@G@CPB) functionalized CoPt@G platform for targeted and selective combating *H. pylori* infection. The CoPt@G shows superior stability in acidic conditions due to the protection of the graphitic shell, and its oxidase‐like activity is activated to catalyze the generation of superoxide radical species for antibacterial applications, and meanwhile, its oxidase‐like activity is suppressed under intestinal neutral conditions. In the *H. pylori*‐infected mouse model, CoPt@G@CPB shows high antibacterial activity toward *H. pylori* and minimal toxicity on intestinal symbiotic bacteria and normal tissues. In addition, MRI and Raman imaging are used to monitor the distribution of CoPt@G@CPB in vivo to guide treatment and follow‐up (Figure 7B). In conclusion, CoPt@G@CPB with in vivo activated oxidase‐like activity shows great promise in future clinical treatment of *H. pylori* infections.

## 4 CHALLENGES AND FUTURE PERSPECTIVES

Metal graphitic nanocapsules have been demonstrated as promising platforms for bioanalysis, bioimaging, and therapy applications due to their superior biocompatibility, excellent stability, and unique physicochemical properties. Despite metal graphitic nanocapsules having their distinctive advantages, there are still some noticeable blocks that need to be solved urgently.

### 4.1 Precise preparation and rational functionalization

Core–shell structured metal graphitic nanocapsules with different compositions, sizes, and morphologies have been prepared by simple CVD method, but thickness of the graphitic shells is difficult to be precisely controlled. There is no doubt that strict control of the size and graphitic layer's thickness of metal graphitic nanocapsules can improve the accuracy of bioanalysis. In particular, SERS intensities decrease significantly with the increase of the distance between the SERS substrate and analyte according to the electromagnetic enhancement mechanism,^[28a,28d^ ^]^ indicating synthesis of PGNs and MPGNs with the external shell of one‐layer graphene facilitates sensitive SERS bioanalysis. Similarly, better magnetic properties of MGNs and obviously enhanced catalytic activity of ELGNs can also be obtained by decreasing their graphitic shells’ thickness and more rational modification. Currently, metal graphitic nanocapsules are commonly functionalized with C_18_‐PEG via hydrophobic interactions to improve water solubility and biocompatibility.^[4c^ ^]^ However, such noncovalent strategy makes the C_18_‐PEG binding unstable on metal graphitic nanocapsules surface and probably causes their aggregation in physiological conditions. Moreover, the long‐chain structured C_18_‐PEG reduces the immobilization efficiency of targeting or drug molecules on metal graphitic nanocapsules surface and hinders the analytes contact with the SERS substrate. The problems above probably limit their extensive applications in biomedicine. Hence, it is urgently needed to improve preparation method and propose more rational functionalization strategy to obtain more ideal metal graphitic nanocapsules for meeting the requirements of biomedical applications.

### 4.2 Toxicity and biosafety

Undoubtedly, it is very important to understand the toxicological characteristics and metabolic behavior of metal graphitic nanocapsules in biological systems before clinical applications. Like other graphitic nanomaterials, biocompatibility of metal graphitic nanocapsules is related to their in vivo degradation and organ distribution.^[^ ^71^ ^]^ Amphipathic C_18_‐PEG is commonly used to functionalize metal graphitic nanocapsules via hydrophobic interactions for improved biocompatibility.^[4c^ ^]^ A series of studies have shown that diverse metal graphitic nanocapsules within a certain dosage range have negligible inhibitory effect on the proliferation of different cell lines, indicating good biocompatibility.^[^ ^13^, ^26^, ^27^ ^]^

In the AuNR@G‐P‐aspirin injected mice model via tail vein, the AuNR@G mainly accumulates in the tumor, liver, and spleen, indicating that the AuNR@G is removed by the liver and spleen metabolism. Histology analysis indicates AuNR@G has no obvious potential toxic and side effects to the main organs of mice, indicating the tumor targeting ability of AuNR@G and its low toxicity at the tested dosage.^[15b^ ^]^ Similar results have been obtained in the CoPt@G injected mice model via tail vein.^[^ ^16^ ^]^ In addition, oral administration of an appropriate concentration of FeCo@G or CoPt@G into *H. pylori*‐infected mice body has no significant damage to the cell morphology, gastric mucosa, and main organs in a short time, and most of them have been excreted from the body within 12 h.^[^ ^13^, ^14^, ^17^ ^]^ However, the long‐term toxicity in the organism of metal graphitic nanocapsules via tail vein or oral administration is required to be systematically explored.

### 4.3 Future perspectives

To date, versatile metal graphitic nanocapsules have made its mark in biomedicine including biodetection, bioimaging, and therapy, but it is still challenged to meet the applications in clinical biomedicine. Metal graphitic nanocapsules have been used for in vivo theranostic of diseases in mice model, but the applications in large animal models have not yet involved and the long‐term toxicity in the mice's organism via tail vein or oral administration is ignored. Hence, our follow‐up work will be committed to exploring the long‐term toxicity of metal graphitic nanocapsules on organism and expanding their application to large primates. Much more efforts must be made to realize diseases theranostic of human beings due to the huge difference in physiological characteristics between humans and mice. We will try our best to advance the development of metal graphitic nanocapsules in future clinical applications. In addition, it is very important to develop novel metal graphitic nanocapsules with richer expected functions for broadened biomedical applications, such as anisotropic PGNs with strong absorption in the NIR‐II region and novel metal graphitic nanocapsules with both plasmonic properties and catalytic activity.

## 5 CONCLUSIONS

Core–shell structured metal graphitic nanocapsules have attracted extensive interest in biomedicine due to their superior stability, good biocompatibility, and unique physicochemical properties. In this review, we first introduce the strategies for metal graphitic nanocapsules synthesis, in which CVD method confers advantages for the preparation of high‐quality metal graphitic nanocapsules. Taking the fumed SiO_2_ template mediated CVD method as an example, the specific growth mechanism is discussed. Second, we introduce the representative properties and applications of the versatile metal graphitic nanocapsules. According to the composition and properties of the metal core, the metal graphitic nanocapsules are divided into four types including PGNs, MGNs, MPGNs, and ELGNs, and their unique properties are introduced in detail. Due to their unique physicochemical properties, the metal graphitic nanocapsules show great promise in biomedicine, and the latest research progress in biodetection, bioimaging, and therapy are systematically described. Eventually, the underlying challenges and development prospects of metal graphitic nanocapsules are discussed adequately. We expect the versatile metal graphitic nanocapsules will offer robust nanoplatforms for future clinical theranostics.

## CONFLICT OF INTEREST

The authors declare no conflict of interest.
